# Editorial: Exposure to Endocrine-Disrupting Chemicals and Cardiometabolic Disease: A Developmental Origins Approach

**DOI:** 10.3389/fpubh.2019.00288

**Published:** 2019-10-09

**Authors:** Wei Perng, Jaclyn M. Goodrich, Andres Cardenas, Deborah J. Watkins

**Affiliations:** ^1^Lifcourse Epidemiology of Adiposity and Diabetes (LEAD) Center, University of Colorado Anschutz Medical Campus, Aurora, CO, United States; ^2^Department of Epidemiology, Colorado School of Public Health, Aurora, CO, United States; ^3^Department of Nutritional Sciences, University of Michigan School of Public Health, Ann Arbor, MI, United States; ^4^Department of Environmental Health Sciences, University of Michigan School of Public Health, Ann Arbor, MI, United States; ^5^Division of Environmental Health Sciences, School of Public Health, University of California, Berkeley, Berkeley, CA, United States

**Keywords:** Developmental Origins of Health and Disease (DOHaD), endocrine-disrupting chemical, obesity, cardio-metabolic abnormalities, children, adolescents

Over the latter half of the twentieth century, community-level exposure to mercury-contaminated grain in Iraq and seafood in Japan exemplified the toxic consequences of environmental pollutants on fetal brain development (1). Since these devastating historical events, researchers have uncovered detrimental effects of toxicants even at low-levels of exposure (2, 3). Many of these toxicants are thought to act by disrupting the normal biosynthesis, metabolism, or action of hormones in the body, and are thus referred to as Endocrine Disrupting Chemicals (EDCs).

In parallel with interest in the role of EDCs in disease etiology, a trio of studies published in the Lancet in the late 1980s/early 1990s put forth the hypothesis that environmental exposures during vulnerable developmental stages—the *in utero* period in particular—have a permanent impact on an organism's metabolic phenotype (4–6). What was initially referred to as “Barker's hypothesis” subsequently expanded into the “Developmental Origins of Health and Disease (DOHaD) hypothesis” which posits that environmental exposures during multiple sensitive periods of development (not just the *in utero* period) have a lasting impact on health and disease risk (7).

This Research Topic places the fields of environmental epidemiology and exposure assessment within the DOHaD framework. We received four articles that provide novel insights into the effect of environmental exposures during sensitive periods of development on cardiometabolic traits across childhood and adolescence:

Vafeiadi et al. examined associations of phthalate exposure during gestation and childhood (~4 years of age) with adiposity and other cardiometabolic traits in 500 Greek children in the Rhea mother-child cohort. While the authors did not find any consistent associations of prenatal phthalate exposure with adiposity or metabolic risk at age 4 years, they detected an inverse association between childhood exposure to certain phthalate metabolites—namely, MEP, MnBP, MBzP—with blood pressure, and a positive association of MiBP with cholesterol; as well as sex-specific associations of concurrent phthalate exposure with adiposity indicators.

Using a similar study design, Moynihan et al. investigated associations of prenatal and concurrent cadmium exposure on adiposity at age 8–14 years among 185 participants in the Early Life Exposure in Mexico to ENvironmental Toxicants (ELEMENT) Project. The authors reported an inverse relationship between prenatal cadmium exposure and adiposity during late childhood/early adolescence—an association that was driven by females.

In another study utilizing the ELEMENT cohort, Bowman et al. explored associations between trimester-specific and adolescent phthalate exposure with adiposity at two time-points across the pubertal transition among 109 boys and 114 boys, and assessed DNA methylation as a potential mediator to these relationships. Exposure to certain phthalates (MBP and MiBP) during early gestation was associated with an increase in adiposity among girls, whereas concurrent exposure to MBzP was associated with decreased adiposity among boys. The investigators noted differential DNA methylation of two growth-related genes (*H19* and *HSD11B2*) in relation to prenatal and adolescent phthalate exposure, as well as with respect to adiposity across adolescence, suggesting involvement of these genes in associations of interest. Mediation analysis did not reveal any statistically significant pathways; however the analysis was likely underpowered. Future, larger studies exploring epigenetic mechanisms as a mediator between early life environmental exposures and childhood outcomes are warranted.

In a methodological study, Zhou et al. leveraged targeted metabolomics analyses in plasma and urine of 115 pregnant women at ~26 weeks gestation to identify novel metabolomics biomarkers of phthalate exposure, and to gain insight into perturbed biochemical pathways. The authors identified metabolites in both plasma and urine that suggested the involvement of diverse pathways, including enhanced lipid, steroid, and nucleic metabolism, and an upregulated inflammatory response in relation to higher phthalate concentrations in both plasma and urine.

Beyond the work carried out by contributors to this Research Topic, future research in this field is needed to interrogate the effects of toxicant mixtures, which more accurately reflect real-life exposure than single toxicants or summary scores of multiple toxicants; the interactive effects of toxicant exposure during multiple sensitive periods throughout the life course, which reflects accumulation of risk; and studies that consider multiple mediating mechanisms, including but not limited to epigenetics, proteomics, metabolomics, and the microbiome to gain a more comprehensive understanding of mechanistic pathways that not only link environmental exposures to adverse health outcomes but also influence susceptibility to toxicity from these exposures.

## Author Contributions

WP conceived the idea for the editorial, wrote the initial draft, and incorporated comments from co-editors. JG, AC, and DW provided critical feedback. All authors approved the final version of the editorial.

### Conflict of Interest

The authors declare that the research was conducted in the absence of any commercial or financial relationships that could be construed as a potential conflict of interest.
